# War and Migration – when Mental Health is left behind

**DOI:** 10.1192/j.eurpsy.2024.1278

**Published:** 2024-08-27

**Authors:** A. L. Ramos, H. Salgado

**Affiliations:** ^1^Psychiatry, Faculty of Medicine University of Oporto; ^2^Psychiatry, University Hospital Center of São João, Oporto, Portugal

## Abstract

**Introduction:**

Wars and armed conflicts are known to have devastating consequences for both physical and mental health of all the people involved. Studies have shown that conflict situations cause more mortality and disability than any major disease and, among the consequences of war, the impact on mental health of the civilian population is one of the most significant.

Forced migration, compelling people to become internally displaced or refugees who have fled to other countries, is responsible for additional physical and mental health problems. Regardless of the reasons for migration, the process itself can be a highly stressful life event, leading to a higher risk of psychiatric disorders. Refugees are particularly susceptible to mood and anxiety disorders, whose prevalence rates is almost twice as high as those found among non-refugee migrants.

**Objectives:**

Since 2022, with the progression of the conflict between Russia and Ukraine, and the establishment of a real war scenario, many Ukrainians were forced to leave their homeland, to ensure their survival and security. In Europe, many countries took in Ukrainian refugees and Portugal was no exception.

In the Psychiatry Inpatient Service of University Hospital Center of São João, there were admissions of Ukrainian refugees who already had a known mental disease - at that time decompensated - and also new cases, to date without follow-up by the specialty.

**Methods:**

In this work, we will carry out a bibliographical review on the impact of war and migration on mental health and the potential of proper medical approach, based on articles indexed in Pubmed, in the last 10 years.

Furthermore, we will present the cases of war refugees interned in our service between January 2022 and December 2023.

**Results:**

We will describe the psychopathological features and also the sociofamilial circumstances of these patients, as well as explain the intervention and longitudinal support developed in these cases.

**Conclusions:**

As a conclusion, we point out the importance of approaching mental illness in light of the individual’s context, knowing that this context may contain the problem and also the solution. War and forced migration bring increased challenges to psychiatry and, in an increasingly globalized society, geographical, linguistic or cultural barriers cannot impose limits on our best and most appropriate medical treatment.

**Disclosure of Interest:**

None Declared

